# Unhealthy diets increase the likelihood of being overweight or obese among African migrant students in China, but not among African non-migrant students: a cross-sectional study

**DOI:** 10.3389/fnut.2024.1291360

**Published:** 2024-03-15

**Authors:** Doris Abra Awudi, Anita Nyarkoa Walker, Mary Makhala Weeto, Christiana Babymay Priddy, Otobong Donald Akan, Cynthia Anuseh Baduweh, Bella Abigail Arthur, Salimata Yakubu, Solim Essomandan Clémence Bafei, Timothy Mobolaji Olagunju, Margaret Zaitoun, Yuxia Zhong, Yucong Feng, Yuandie Zhang, Tao Wei, Qing Feng

**Affiliations:** ^1^Department of Nutrition and Food Hygiene, Nanjing Medical University, Nanjing, Jiangsu, China; ^2^Microbiology Department, Akwa-Ibom State University, Uyo, Akwa-Ibom State, Nigeria; ^3^Food Science and Engineering, Central South University of Forestry and Technology, Hunan, China; ^4^School of Public Health, Nanjing Medical University, Nanjing, Jiangsu, China; ^5^School of Medicine, Jiangsu University, Zhejiang, Jiangsu, China; ^6^Department of Epidemiology and Health Statistics, Nanjing Medical University, Nanjing, Jiangsu, China

**Keywords:** global diet quality score, overweight or obesity, eating habits, Africans, western diet, China, students, diet quality

## Abstract

**Background:**

The impact of non-communicable diseases (NCDs) is disproportionately felt by immigrants from low- to medium-income countries (LMICs), partly due to their dietary habits. To thrive in their new environment, migrants either omit or consume certain food items, which could lead to nutritional deficits. As a result, most migrants experience more NCDs than their compatriots in their native countries. Therefore, we evaluated the difference in dietary habits, quality, and the influencing factors of overweight or obesity among African migrant students in Nanjing (China) and non-migrant students in Africa using cross-sectional data.

**Methods:**

The researchers used the food frequency questionnaire and the global diet quality score metrics to assess food intake and quality, respectively. Then, cross-tabulation was employed to explore the differences between the groups in meal skipping, eating habits, and diet quality. Finally, the factors associated with overweight or obesity were assessed with binary logistic regression stratified by African students in Nanjing and students in their native countries.

**Results:**

Approximately 678 responses were received, mainly between 18–25 years (46.7%) and 26–36 years (45.4 %). The majority of them (52.3%) were international students. The non-migrant African students' diets lacked citrus fruits (22.2%), deep orange fruits (15.4%), deep orange vegetables (18%), cruciferous vegetables (24.6%), and dark leafy vegetables (26.5%). While the African migrant students consumed more high-fat dairy (50.7%), processed meats (23.9%), sweets and ice creams (51.3%), sugar-sweetened beverages (40.5%), and juice (61.5%), *p* < 0.001. Furthermore, consuming late-night meals constantly [Exp (B) = 39.607, *p* = 0.049], eating twice a day [Exp (B) = 6.527, *p* = 0.036], consuming red meat [Exp (B) = 29.287, *p* = 0.001], processed meats [Exp (B) = 719.979, *p* = 0.0011], refined grains and baked foods [Exp (B) = 15.752, *p* = 0.013], and sweets and ice cream [Exp (B) = 193.633, *p* = 0.006] were factors inducing overweight or obesity among only African migrant students.

**Conclusion:**

Controlling the what (Western diet and nature of late-night meals) and the when of eating can drastically reduce their influence on obesogenic condition formation in African migrant students in China and elsewhere.

## 1 Introduction

Migrating from one's country of origin to another might instigate changes in behavior and lifestyle. Food is a critical aspect of cultures; therefore, immigration can drastically change an individual's survival core (1). Interestingly, dietary complications among migrants are mainly the result of changes in diet. A mediating element for the high frequency of nutritional disorders among migrants, besides their low economic situation, is insufficient or lack of knowledge regarding local foods (2, 3). To thrive in their new environment, migrants either omit or consume certain food items, which could lead to nutritional deficits (4). For instance, according to the study by Zhu et al., Chinese international students in South Korea consumed fewer fruits and vegetables, more readymade foods, and mostly missed breakfast (5). However, unhealthy diets and practices such as consuming sugary drinks, fast foods, and fewer fruits and vegetables, skipping meals, particularly breakfast, and binge eating, among others, contribute to weight gain and, as a result, increase the burden of non-communicable diseases (NCDs) globally (6).

Non-communicable diseases, such as overweight or obesity, are prime culprits in a pandemic across the globe. Aside from infectious and chronic diseases being burdensome to people of African origin, with at least 69% of fatalities from infectious diseases, current projections suggest increased death rates from diabetes, cancer, respiratory, and cardiovascular disease-induced NCDs among Africans in the next 10 years (7). Additionally, NCD impacts are disproportionately felt by immigrants from lower-middle-income countries (LMICs) (8). According to a study among African Migrants in Europe, diabetes, obesity, and higher CVD risk are becoming more common among Europe-based Ghanaian migrants compared to their Ghana-based fellow citizens (9, 10). However, observable disparities in diet-induced health issues among immigrant and non-immigrant counterparts in their home countries are not just among Africans. For instance, when consuming foods rich in fats, Chinese living in America also have a greater incidence of long-term illnesses such as excessive weight gain, hypertension, sugar diseases, disease of the heart, and some types of malignancies than Chinese living in China. Suggesting that migration might be a culprit for these health inequalities (11).

The specific nature of food culture for a particular group of people (12) can also account for the dietary problems resulting in NCDs among migrants. For instance, there is a significant difference in the availability of African staple foods such as millet, sorghum, plantains, and cassava, among others, compared to the staples of Asians such as rice and wheat (13). The differences in diet availability pose a severe problem for most African migrants outside of Africa, making them skip meals and consume excessive amounts of fast food. The Pew Research Center has estimated that in 2010, African migrants comprised 80% of all international migrants in other countries, including China (14). However, no study has examined the diet quality of immigrant African students in China and how it affects their weight status. This study's outcome can inform decision-makers and migrant students on how to manage overweight or obesity risks among African students in China.

## 2 Materials and methods

### 2.1 Study design, population, and sampling

This study examined the quality of African international students' diets in Nanjing and compared them with those of their compatriot students in their respective African countries. The differences in influencing factors of overweight or obesity across the two groups were also assessed using a cross-sectional study. A purposive and convenient sampling technique was employed to recruit African students in Nanjing, Lesotho, Ghana, and Nigeria. Nanjing is located in the east of China, hosting most international students in Jiangsu province since it is the province's capital and second-largest city. The students from Lesotho attended the National University of Lesotho, situated in the capital of Lesotho. The Ghanaian students were from the University of Cape Coast in Cape Coast, Ghana. Finally, the Nigerian students were from the University of Lagos in Akoka, Nigeria. This study employed an online data collection method for international students in Nanjing and a face-to-face interview for African students in their respective countries. The researchers pretested the questionnaire with ten students from Nanjing Medical University, China; the University of Cape Coast, Ghana; the University of Lagos, Nigeria, and the National University of Lesotho, Lesotho. The final version was then hosted on Wenjuanxing for international students in China and on Google Forms for non-international students.

The study included only international African students in Nanjing who accepted to participate. At the same time, the non-international students comprised only students from the selected schools who decided to participate in the survey. Data collection took place between 1 August and 30 December 2022. We provided no incentive for participation. No personal identification information (confidentiality) was collected. The participants could complete the survey within 10–15 min. All study participants provided their consent before participating by selecting “yes” to continue with the study or “no” to withdraw from the study. The School of Public Health, Nanjing Medical University's research committee reviewed the study protocol. However, the ethics committee of Nanjing Medical University exempted the study from obtaining ethical approval because it did not collect biological samples or receive confidential information. Nevertheless, Helsinki's ethical standards and later amendments were adhered to. Furthermore, the heads of schools and departments of the various universities and colleges of the universities in Africa gave their consent before the researchers collected the data from the different schools.

### 2.2 Dietary intake assessment

Researchers used a proven semi-quantitative food frequency questionnaire (FFQ) specific to our populations (15–19) to access the food consumption of the respondents – the final tool comprised 100 food items for each country. The frequency of consuming food was estimated as “four to six times per day,” “two to three times per day,” “once every day,” “two to six times per week,” “once every week,” “once to three times per month,” and “never.” Specific portion sizes were added to the questionnaire to ensure that the respondents selected the actual amount they consumed. For each frequency option, we used the following values: never = 0; once to three times per month = 2/30; once every week = 1.5/7; two to six times per week = 4/7; once every day = 1; two to three times per day = 2.5; and four to six times per day = 4. Next, the research team estimated the mass consumed per day by multiplying the corresponding value of the frequency of consuming food items by the standard portion size (20).

### 2.3 Diet quality assessment

We used the Global Diet Quality Score (GDQS), a food-based dietary quality score, to determine the adequacy of our respondents' dietary intake. This index was developed based on the Prime Diet Quality Score. The GDQS aims to explore dietary quality and investigate how the food we eat relates to long-term diseases across the globe. The GDQS includes 25 food groups: 16 healthy food groups, 2 unhealthy groups when consumed in excess, and 7 unhealthy food groups. The detailed components of the food groups are published elsewhere (21). The scoring of the GDQS is based on the quantity of food consumed daily. Positive scores are given to the healthy 16 food groups, and higher scores denote higher consumption. Next, for the unhealthy food groups, except high-fat dairy and red meat, moderate consumption received a higher score, whereas very low or extremely high intakes received a worse score. A higher score on the GDQS, which ranges from 0 to 49 points, indicates higher dietary quality. The GDQS negative (GDQS–) sub-metric is composed of the addition of the score of the unhealthy food categories and ranges from 0 to 17 points, whereas the GDQS positive (GDQS+) sub-metric is composed of the addition of the score of the healthy food groups and ranges from 0 to 32 points (21).

### 2.4 Eating habits

This aspect was constructed based on existing literature about practices recognized as healthy or unhealthy eating behaviors. The behaviors examined include fast food consumption, binge eating, late-night meals, healthy meal choices, reading food labels, and consuming soda and alcoholic beverages. The responses to these questions were “never,” “sometimes,” “most of the time,” and “always.” This aspect also consisted of whether they skipped meals, the type of meal skipped, and the number of times they ate in a day.

### 2.5 Other variables

This component was added to collect information on demographic and anthropometric variables. Age, sex, continent of origin, educational level, present residence, and average monthly expenses were among the demographic variables.

The weight (kg) and height (m) of African students in Nanjing were self-reported. However, using a weighing scale, the data collectors weighed the non-international African students in their home nations while dressed in loose clothing and without shoes. We reported their weight and height to be closest to 0.5 kg (1.1 pounds). Before using any measurement equipment for the first time, they calibrated it to ensure that the collected data was accurate. We then calculated their body mass index (BMI) in kg/m^2^ by dividing their weight by the square of their height. We followed the recommendations of the National Institute of Health to categorize the BMI. Here, underweight is defined as having a BMI of 18.5 or less. A BMI between 18.5 and 24.9 is considered average weight, and a BMI between 25 and 29.9 is defined as overweight while a BMI of 30 or more is defined as obese (22).

### 2.6 Statistical analyses

The Statistical Package for Social Sciences was used for the analysis (version 26; SPSS Inc., Chicago, IL). The data were described through frequencies, percentages, means, and standard deviations. Then, cross-tabulation was used to determine how meal skipping, eating habits, and diet quality differed among the students in Nanjing and the students in their native countries. The predisposing factors of overweight or obesity were assessed with binary logistic regression stratified by African students in Nanjing and African students in their native countries, with weight status as the outcome variable and diet quality and eating habits as the explanatory variables. All statistical analyses were conducted with a significance level of *p* < 0.05 Table 1.

**Table 1:** How meal-skipping habits vary across African international students in Nanjing and non-international African students in their native countries.

**Variables**	**Response**	**African international students in Nanjing**	**Non-international African students in their native countries**	**Pearson chi-square**	***P*-value**
Do you skip meals frequently?				5.001	0.025
	Yes	215 (62.7)	176 (54.2)		
	No	128 (37.3)	149 (45.8)		
What type of meal do you skip?					
	I don't skip any meal	128 (37.3)	149 (45.8)	57.407	< 0.001
	Breakfast	179 (52.2)	87 (26.8)		
	Lunch	26 (7.6)	75 (23.1)		
	Supper	10 (2.9)	14 (4.3)		
How often do you eat daily?				79.895	< 0.001
	Once	8 (2.3)	5 (1.5)		
	Twice	217 (63.3)	96 (29.5)		
	Thrice and more	118 (34.4)	224 (68.9)		

## 3 Results

We received a total of 678 responses, and 53% were received from women. The bulk of respondents were between the ages of 18 and 25 years (46.7%) and 26 and 36 years (45.4%) and were primarily undergraduates (53.4%). They consisted of African students in Nanjing (52.3%) and African students from Lesotho (29.9%), Nigeria (10.5%) and Ghana (8.2%). Finally, the majority of students were healthy (55.7%), while approximately 38.7% were overweight or obese (Table 2).

**Table 2:** The demographic distribution of the students.

		**Total**	**Migrant students in Nanjing**	**Non-migrants**
**Variables**	**Subgroups**	**Frequency (percentage)**	**Frequency (percentage)**	**Frequency (percentage)**
**Sex**				
	Male	324 (47.0)	182 (53.1)	132 (40.6)
	Female	354 (53.0)	161 (46.9)	193 (59.4)
**Age**				
	Between 18 and 25 years	312 (46.7)	94 (27.4)	218 (67.1)
	Between 26 and 35 years	303 (45.4)	209 (60.9)	94 (28.9)
	36 years and above	53 (7.9)	40 (11.7)	13 (4.0)
**Educational level**				
	Undergraduates and below	357 (53.4)	99 (28.8)	258 (79.4)
	Postgraduates and above	311 (46.6)	244 (71.2)	67 (20.6)
**Weight status**				
	Underweight	37 (5.5)	5 (1.5)	32 (9.8)
	Healthy weight	372 (55.7)	209 (60.9)	163 (50.2)
	Overweight	190 (28.4)	98 (28.6)	92 (28.3)
	Obese	69 (10.3)	31 (9.0)	38 (11.7)
**Current residing location**				
	African students in Nanjing	342 (52.3)		
	Ghanaians			156 (45.61)
	Togolese			58 (16.96)
	Nigerians			44 (12.87)
	Zimbabwean			6 (1.75)
	Liberian			6 (1.75)
	Tanzanian			9 (2.63)
	Mauritian			5 (1.46)
	Kenyan			4 (1.17)
	Cameroon			8 (2.34)
	Ugandan			3 (0.88)
	Sierra Leonean			3 (0.88)
	Motswana			3 (0.88)
	Benin			3 (0.88)
	Botswana			3 (0.88)
	Lesotho			29 (8.48)
	Mauritius			2 (0.58)
African students in their respective countries		325 (48.6)		
	Ghanaians		55 (16.92)	
	Nigerians		70 (21.53)	
	Lesotho		200 (61.54)	

A more significant number of African students in Nanjing (62.7%) skipped meals (*p* = 0.025), especially breakfast (52.2%), *p* < 0.001. The results show that most African students (64.9%) at home eat thrice (and more) a day, while African students in Nanjing (63.3%) mostly eat twice a day (*p* < 0.001).

The eating habits of African students in Nanjing were found to be less healthy compared to their counterparts in their native countries. The students in Nanjing frequently (93.7%) consumed fast foods such as burgers, pizza, and fries. Additionally, it was found that more international students in Nanjing often consume food during late hours (68.9%) and binge eat (64.5%). However, the late-night meals eaten by a majority of African students in Nanjing were considered healthy (74.5%); they regularly read the food labels of the food they consume (86.3%), This difference was statistically significant (*p* < 0.001) (Table 3).

**Table 3:** How eating habits vary across African international students in Nanjing and non-international African students in their native countries.

**Variable**	**African international students in Nanjing**			**Non-international African students in their native countries**			**Chi-square**	***P*-value**
	**Never**	**Sometimes**	**Most of the time**	**Never**	**Sometimes**	**Most of the time**		
How frequently do you eat fast food, such as burgers, pizza, fries, etc.?	27 (7.9)	281 (81.9)	35 (10.2)	15 (6.3)	221 (75.1)	124 (18.6)	33.65	< 0.001
How frequently do you eat something after 10 pm?	30 (8.7)	201 (58.6)	112 (32.7)	101 (31.1)	142 (43.7)	82 (25.2)	52.82	< 0.001
When are these late-night snacks and meals healthy?	68 (19.8)	144 (42.0)	131 (38.2)	118 (36.3)	147 (45.2)	60 (31.4)	39.41	< 0.001
I select healthy foods when offered to eat fast food or healthy meals.	7 (2.0)	41 (28.1)	295 (86.0)	14 (4.3)	105 (32.3)	206 (63.4)	45.75	< 0.001
How frequently do you drink soda/alcoholic beverages?	108 (31.5)	195 (56.9)	40 (11.7)	83 (25.5)	179 (47.9)	63 (19.4)	8.614	0.013
How often have you found yourself binge-eating?	122 (35.6)	146 (42.6)	75 (21.9)	147 (45.2)	132 (40.6)	46 (14.2)	9.501	0.009
How probable is it that you will consider the nutrition label of food items before eating them?	45 (13.1)	134 (39.1)	164 (47.8)	107 (32.9)	132 (40.6)	86 (26.5)	49.19	< 0.001
How frequently do you eat in the cafeteria or restaurant?	25 (7.3)	235 (68.5)	83 (24.2)	38 (11.7)	217 (66.8)	70 (21.5)	4.022	0.134

For each of the 25 food groups in the GDQS, the average amount consumed is shown in Table 4. The quality of the diet of the students varied significantly across the African students in Nanjing and their counterparts in their respective countries. Here, the diets of African students in their respective countries suffered more from inadequate intake of citrus fruits (22.2%), deep orange fruits (15.4%), deep orange vegetables (18%), cruciferous vegetables (24.6%), and dark leafy vegetables (26.5%). Moreover, the African international students in Nanjing's consumption of legumes (47.8%) and fish and shellfish (44%) were inadequate. On the other hand, they also consumed excessive amounts of high-fat dairy (50.7%), processed meats (23.9%), sweets and ice creams (51.3%), sugar-sweetened beverages (40.5%), and juice (61.5%), and purchased deep fried foods (11.9%), *p* < 0.001.

**Table 4:** The mean consumption for each of the 25 GDQS food groups.

**GDQS component**	**Mean (S.E.)**	**Total**			**African international students in Nanjing**			**Non-international African students in their native countries**			**Chi-square**	***P*-value**
		**Lower *n* (%)**	**Moderate *n* (%)**	**Higher *n* (%)**	**Lower *n* (%)**	**Moderate *n* (%)**	**Higher *n* (%)**	**Lower *n* (%)**	**Moderate *n* (%)**	**Higher *n* (%)**		
Citrus fruits	164.61 (9.55)	119 (17.8)	143 (21.4)	406 (60.8)	47 (13.7)	105 (30.6)	191 (55.7)	72 (22.2)	38 (11.6)	215 (66.2)	37.61	< 0.001
Deep orange fruits	272.17 (15.53)	88 (13.2)	233 (34.9)	347 (51.9)	38 (11.1)	93 (27.1)	212 (61.8)	50 (15.4)	140 (43.1)	135 (48.7)	27.74	< 0.001
Other fruits	325.28 (23.03)	175 (26.2)	183 (27.4)	310 (46.2)	88 (25.7)	101 (29.4)	154 (52.2)	87 (27.7)	82 (26.1)	145 (47.8)	0.971	0.615
Dark green leafy vegetables	124.49 (8.50)	117 (17.5)	76 (11.4)	475 (71.1)	31 (9.0)	76 (22.2)	236 (68.8)	86 (26.5)	0 (0.0)	239 (73.6)	387.85	< 0.001
Cruciferous vegetables	111.87 (5.20)	94 (14.1)	127 (19.0)	447 (66.9)	14 (4.1)	62 (18.1)	267 (77.8)	80 (24.6)	65 (20.0)	180 (55.4)	62.91	< 0.001
Deep orange vegetables	131.87 (9.06)	65 (9.7)	207 (31.0)	396 (59.3)	6 (1.7)	93 (27.1)	244 (71.1)	59 (18.2)	114 (35.1)	152 (46.8)	66.28	< 0.001
Other vegetables	344.12 (14.87)	9 (1.3)	67 (10.0)	592 (88.6)	3 (0.9)	30 (8.7)	310 (90.4)	6 (1.8)	37 (11.4)	282 (86.8)	2.573	0.276
Legumes	108.48 (10.44)	243 (36.4)	197 (29.5)	228 (34.1)	164 (47.8)	109 (31.8)	70 (20.4)	79 (24.3)	88 (27.1)	158 (48.6)	65.50	< 0.001
Deep orange tubers	81.07 (4.20)	209 (31.3)	176 (26.3)	283 (42.4)	15 (4.4)	143 (41.7)	185 (53.9)	194 (59.7)	33 (10.2)	98 (32.2)	248.50	< 0.001
Nuts and seeds	144.70 (11.66)	146 (21.9)	15 (2.2)	507 (75.9)	70 (20.4)	15 (4.4)	258 (93.1)	76 (23.4)	0 (0.0)	249 (77.7)	451.30	< 0.001
Whole grains	757.95 (30.69)	2 (0.3)	0 (0.00)	666 (99.7)	2 (0.6)	0 (0.0)	341 (99.4)	0 (0.0)	0 (0.0)	325 (100.0)	1.90	0.168
Liquid oils	29.55 (1.66)	50 (7.5)	78 (11.7)	540 (80.8)	30 (8.7)	59 (17.2)	254 (51.3)	20 (6.2)	19 (5.8)	286 (88.0)	23.94	< 0.001
Fish and shellfish	80.69 (6.40)	280 (41.9)	216 (32.3)	172 (25.7)	151 (44.0)	109 (31.8)	83 (24.2)	129 (39.7)	107 (32.9)	89 (27.4)	1.47	0.479
Poultry and game meat	235.13 (13.21)	49 (7.3)	84 (12.6)	535 (80.1)	9 (2.6)	32 (9.3)	302 (88.0)	40 (12.3)	52 (16.0)	233 (71.7)	32.81	< 0.001
Low-fat dairy	81.16 (5.77)	408 (61.1)	156 (23.4)	104 (15.6)	185 (53.9)	117 (34.1)	41 (12.0)	223 (68.6)	39(12.0)	63 (19.4)	46.74	< 0.001
Eggs	45.46 (1.85)	53 (7.9)	240 (35.9)	375 (56.1)	22 (9.6)	191 (83.4)	16 (7.0)	31 (9.5)	126 (38.8)	168 (51.7)	127.62	< 0.001
High-fat dairy	259.85 (12.62)	215 (32.2)	129 (19.3)	324 (48.5)	86 (25.1)	83 (24.2)	174 (50.7)	129 (39.7)	46 (14.2)	150 (46.2)	20.52	< 0.001
Red meat		375 (56.7)	273 (40.9)	16 (2.4)	172 (50.1)	155 (45.2)	16 (4.7)	207 (63.7)	118 (36.3)	0 (0.0)	23.78	< 0.001
Processed meat	141.69 (11.56)	185 (27.7)	334 (50.0)	149 (22.3)	0 (0.0)	261 (76.1)	82 (23.9)	185 (56.9)	73 (21.9)	67 (20.6)	292.06	< 0.001
Refined grains and baked goods	277.78 (20.18)	235 (35.2)	390 (58.4)	43 (6.4)	0 (0.0)	327 (95.3)	16 (4.7)	235 (72.3)	63 (19.4)	27 (8.3)	416.34	< 0.001
Sweets and ice cream	102.96 (9.303)	263 (39.4)	149 (22.3)	256 (38.3)	120 (35.0)	47 (31.5)	176 (51.3)	143 (44.0)	102 (31.4)	80 (24.6)	57.87	< 0.001
Sugar-sweetened beverages	310.04 (25.17)	250 (37.4)	223 (33.4)	195 (29.2)	73 (21.3)	131 (38.2)	139 (40.5)	177 (54.5)	92 (41.3)	56 (17.2)	84.99	< 0.001
Juice	58.39 (3.37)	47 (7.0)	248 (37.1)	373 (55.8)	1 (0.3)	131 (38.2)	211 (61.5)	46 (14.2)	117 (36.0)	162 (49.8)	49.86	< 0.001
White roots and tubers	142.98 (9.61)	131 (19.6)	409 (61.2)	128 (19.2)	65 (19.0)	194 (56.6)	84(24.5)	66 (36.7)	70 (38.9)	44 (24.5)	22.10	< 0.001
Purchased deep-fried foods	147.37 (8.74)	370 (55.4)	238 (35.6)	60 (9.0)	153 (44.6)	151 (44.0)	39 (11.4)	217 (66.8)	87 (26.8)	21 (6.5)	22.219	< 0.001

According to Table 5, the predisposing factors of overweight or obesity differed between the two groups. Harmful eating habits such as consuming late-night meals always [Exp (B) = 39.607, *p* = 0.049], eating twice a day [Exp (B) = 6.527, *p* = 0.036], consuming red meat [Exp (B) = 29.287, *p* = 0.001], processed meats [Exp (B) = 719.979, *p* = 0.0011], refined grains and baked foods [Exp (B) = 15.752, *p* = 0.013], and sweets and ice cream [Exp (B) = 193.633, *p* = 0.006] all increased the likelihood of being overweight or obese among African international students in Nanjing compared to African students in their home countries. However, there was a decreased risk of becoming overweight or obese when healthy late-night snacks were consumed always [Exp (B) = 0.082, *p* = 0.0021], sometimes [Exp (B) = 0.032, *p* = 0.002], or never [Exp (B) = 0.033, *p* = 0.008]. Furthermore, avoiding cafeteria and restaurant food [Exp (B) = 0.015, *p* = 0.001], avoiding binge eating [Exp (B) = 0.002, *p* = 0.01], avoiding consuming deep-fried foods purchased from a store [Exp (B) = 0.027, *p* = 0.014], and avoiding consuming sugar-sweetened beverages [Exp (B) = 0.0235, *p* = 0.0037] all decreased the likelihood of being overweight or obese. Finally, a higher global diet quality score also reduced the chance of being overweight or obese [Exp (B) = 0.010, *p* = 0.001] among African international students in Nanjing compared to African students in their respective countries.

**Table 5:** The elements that influence our population's chance of being overweight or obese utilizing binary logistic regression.

**Variables**	**African international students in Nanjing**					**Non-international African students in their native countries**				
	**B**	**Exp(B)**	**Sig**.	**95% C.I. for Exp(B)**		**B**	**Exp(B)**	**Sig**.	**95% C.I. for Exp(B)**	
				**Lower**	**Upper**				**Lower**	**Upper**
**Sex (male)**										
Female	−1.071	0.343	0.269	0.051	2.289	0.133	1.142	0.634	0.661	1.974
**Age (18–25 years)**										
26–35 years	−0.928	0.395	0.320	0.064	2.457	−0.480	0.619	0.099	0.350	1.094
36 years and above	−0.297	0.743	0.806	0.070	7.927	−0.540	0.583	0.423	0.156	2.182
**Fast food consumption (never)**										
Sometimes	−1.014	0.363	0.480	0.022	6.034	−0.158	0.854	0.797	0.257	2.834
Always or most of the time	−3.387	0.034	0.106	0.001	2.063	0.536	1.710	0.445	0.432	6.771
**Late-night meal consumption (never)**										
Sometimes	3.011	20.307	0.123	0.443	931.634	0.239	1.270	0.631	0.479	3.367
Always or most of the time	3.679	39.607	0.049	1.018	1,540.700	0.544	1.723	0.298	0.619	4.799
**Healthy late-night meal (never)**										
Always	−2.507	0.082	0.021	0.010	0.688	−0.063	0.939	0.897	0.364	2.425
Sometimes	−3.428	0.032	0.002	0.004	0.271	0.431	1.538	0.276	0.709	3.337
**Choosing healthy food (always)**										
Never	−1.822	0.162	0.314	0.005	5.599	−1.150	0.317	0.086	0.085	1.177
Sometimes	−0.865	0.421	0.454	0.044	4.053	−0.415	0.660	0.189	0.355	1.226
**Drink soda or alcohol (never)**										
Sometimes	−3.410	0.033	0.008	0.003	0.403	−0.401	0.669	0.229	0.348	1.288
Always or most of the time	−2.759	0.063	0.040	0.005	0.883	−1.080	0.340	0.011	0.148	0.781
**Binge eat (never)**										
Sometimes	0.026	1.026	0.979	0.149	7.076	−0.375	0.687	0.186	0.394	1.198
Always or most of the time	−6.114	0.002	< 0.001	0.000	0.046	−0.686	0.503	0.110	0.217	1.167
**Read food labels (never)**										
Sometimes	−1.596	0.203	0.175	0.020	2.039	−0.337	0.714	0.318	0.368	1.383
Always or most of the time	−1.950	0.142	0.090	0.015	1.360	0.181	1.199	0.647	0.551	2.607
**Eat from cafeteria (always)**										
Never	−7.749	0.015	0.001	0.000	0.021	−0.320	0.726	0.546	0.257	2.054
Sometimes	−1.231	0.292	0.215	0.042	2.042	−0.357	0.700	0.349	0.331	1.478
**Number of eating times in a day (thrice and more)**										
Once	−2.960	0.052	0.056	0.002	1.081	−1.117	0.327	0.356	0.031	3.504
Twice	1.876	6.527	0.036	1.133	37.600	0.280	1.323	0.421	0.669	2.616
**Type of meal skipped (none)**										
Breakfast	2.999	20.068	0.139	0.379	1,061.250	−0.584	0.557	0.391	0.147	2.118
Lunch or supper	3.166	23.705	0.169	0.261	2,154.931	−0.295	0.744	0.672	0.190	2.914
**High-fat dairy (low)**										
middle	−1.086	0.338	0.306	0.042	2.698	0.354	1.425	0.400	0.624	3.253
High	−0.284	0.753	0.762	0.120	4.734	−0.583	0.558	0.043	0.318	0.981
**Red meat (low)**										
Middle	3.377	29.287	0.001	4.311	198.977	0.028	1.029	0.925	0.568	1.865
**Processed meat (low)**										
Middle	6.579	719.979	0.011	4.653	111,405.375	−0.336	0.715	0.350	0.354	1.445
**Refined grains and baked foods (low)**										
Middle	2.757	15.752	0.013	1.774	139.863	0.338	1.402	0.339	0.701	2.806
**Sweets and ice cream (low)**										
Middle	−25.04	0.000	0.999	0.000	0.00	−0.034	0.966	0.927	0.466	2.003
High	5.266	193.633	0.006	4.501	8,329.378	−0.471	0.624	0.251	0.279	1.395
**Sugar-sweetened beverages (low)**										
Middle	−0.183	0.833	0.877	0.082	8.421	−0.478	0.620	0.146	0.325	1.181
High	−5.465	0.004	< 0.001	0.000	0.086	−0.388	0.678	0.352	0.300	1.535
**Juice (low)**										
Middle	−3.360	0.035	0.037	0.001	0.815	0.219	1.244	0.614	0.532	2.908
High	−12.29	0.000	1.000	0.000	0.00	0.070	1.072	0.879	0.436	2.635
**Purchased deep-fried foods (low)**										
Middle	−9.575	0.000	1.000	0.000	0.00	−0.176	0.838	0.579	0.449	1.564
High	−3.604	0.027	0.014	0.002	0.479	0.140	1.151	0.798	0.393	3.372
**GDQS (low)**										
High	−9.182	0.010	< 0.001	0.000	0.013	0.083	1.086	0.807	0.559	2.109

## 4 Discussion

This study aimed to investigate whether there was a relationship between the obesity and overweight risk and the dietary quality and eating behaviors of students from a varied population, specifically, African migrant students in Nanjing and African students in their native countries. A cross-sectional study design was utilized in this investigation. The study discovered that African students in Nanjing practice poor eating behaviors more frequently than in their home countries, which affects their risk of being overweight or obese.

Most migrant students, like those in other studies, miss meals, especially breakfast, consume more fast food, and eat at irregular hours (beyond 10:00 pm) (23, 24). The increase level of breakfast skipping can be attributed to the nature of chinese breakfast which is difficult to consume. Students also skip meals due to the stress of studying abroad, especially when learning a new language (25, 26). Notwithstanding, they were always mindful of the food's nutritional information, and their late-night meals were generally healthy. Due to their higher levels of education—the majority of whom were postgraduates and doctoral students as opposed to non-migrant undergraduates—the migrants may exhibit healthier behaviors than their native-born counterparts (27). In addition, the level of nutritional understanding can be increased by extensive nutrition education programs. Developed nations, such as China, continually promote healthy eating habits and encourage their residents to read food labels.

The non-migrant African students' main dietary issue is their lack of fruit and vegetable consumption. Citrus fruits, deep orange vegetables, cruciferous vegetables, and dark green vegetables were among the foods that non-migrants ate less often. Worldwide, reports of young adults who do not eat enough fruit and vegetables are known (28–30). Additionally, as compared to people in other regions with comparable levels of development, Africans spend the most on food, according to data from the African Union Commission (AUC), the United Nations Economic Commission for Africa (UNECA), and the Food and Agriculture Organization (FAO). As a result, they find it challenging to consume nutrient-dense meals due to their higher cost compared to staples such as cereals and starchy roots (31). Despite this, these foods contain several advantages, including dietary fiber, vitamins, minerals, antioxidants, phytoestrogens, and anti-inflammatory agents, essential for a person's overall health and illness prevention (32). Therefore, measures are required to guarantee that most of the population, particularly students, can access these dietary categories.

Furthermore, the migrant students engaged in an excessive amount of Westernized dining. In particular, they bought and consumed a lot of deep-fried items and processed meat, sweets, ice cream, and beverages with added sugar. This trend in meal consumption is similar to that of other migrants, whose main dietary trends after migration included a significant increase in energy and fat intake and a decrease in carbohydrates, along with switching from whole cereals and beans to more refined types of carbohydrates, meat, and dairy products (33). Additionally, the fact that China is more industrialized than the African nations from which the participants came may have contributed to the rise in consumption (34, 35). However, the increase in consumption of this food group among African migrants in China is detrimental to their health and the world as Westernized diets are connected to many ailments, including type 2 diabetes, obesity, cardiovascular disease, high cholesterol, dementia, and attention deficit hyperactivity disorder (ADHD) (36–38). Due to the effects mentioned above, migrant students must be made aware of the need to create a balanced diet free of or low in Western foods to safeguard their health and the environment.

As anticipated, the risk of becoming overweight or obese among the African migrants increased with their increased consumption of red meat, processed meat, refined grains, baked goods, sweets, and ice cream. These foods are high in saturated fats, the primary cause of most non-communicable disorders such as obesity or being overweight, because they increase low-density lipoprotein (LDL-C) levels (39). The intake of such foods and the increased risk for obesity and other diet-related non-communicable diseases are not recent trends among migrants (40–42). The severe impact this diet is having on migrants' health requires immediate action.

Additionally, unhealthy eating patterns, including regularly eating late at night and eating only twice daily, were effective predictors of being overweight or obese. This result is consistent with a recent study, which indicated that having dinner later in the evening increases various factors that can contribute to obesity (43). Furthermore, Brigham and Women's Hospital researchers discovered that eating after midnight decreases calorie expenditure, increases hunger, and alters fat tissue, which might lead to weight gain (44). Eating less frequently is also linked to an irregular eating pattern that may result in weight gain, like an increase in hormones connected to hunger and, eventually, a metabolic disruption that could raise cardiovascular risk (45). Furthermore, the high risk of being overweight or obese among African migrants who eat twice a day can be attributed to breakfast skipping (46). It is vital to remind migrant students that missing meals would reduce the quantity and quality of nutrients they need each day, and eating unhealthy late-night snacks is detrimental to their health.

Interestingly, consistently eating a healthy late-night snack decreased the likelihood of African migrants being overweight or obese. Only the type of diet taken will determine how harmful late-night meals are to health. Research suggests that eating late-night meals rich in protein, fiber, healthy fats, iron, vitamins A, C, E, folate, and probiotics can help prevent weight gain (47). Even though late-night eating has adverse health effects, young people like our participants cannot entirely break the practice. As a result, informing them about healthy late-night snacks to eat is essential. In addition to late-night snacking, people who avoided eating at cafeterias and restaurants were less likely to be obese or overweight. Research shows that dining at restaurants and cafes harms weight loss and BMI (48). This is because restaurant meals tend to be energy-dense, delicious, and frequently paired with sugary drinks. Moreover, these meals are offered at discounted prices, in large portion sizes, and in appealing packaging, all of which encourage the consumption of larger portions (49). Thus, avoiding eating at restaurants or cafeterias is essential to prevent the risk of weight gain.

A higher global diet quality score also decreased the probability of being overweight or obese. A higher GDQS score indicates a better diet, which can help reduce non-communicable nutrition-related diseases (21). According to the GDQS, people are less likely to gain weight when they consume more plant-based foods that are low in processed grains, red and processed meats, and added sugar. Similar to our findings, U.S. women who experienced a rise in the GDQS reduced the risk of obesity and concurrent weight gain (50). Likewise, Mexican women showed a reduced tendency to gain weight and a smaller waist circumference with increased GDQS (51). Therefore, to prevent weight gain among migrant African students in Nanjing that may lead to overweight or obesity, students should be advised to eat meals high in fruits, vegetables, whole grains, and fish and to cut back on refined grains, red and processed meats, added sugar, and carbonated beverages.

This study is the first to compare eating habits, diet quality, and risk factors for overweight or obesity between non-migrant African students in their respective countries and African migrant students in Nanjing (China). It does, however, have several limitations that should be taken into account when evaluating the results. The migrant students in Nanjing self-reported their weight and height, which could lead to recall bias. Nevertheless, previous research has shown that self-reported weight and height are adequate for calculating BMI (52). Second, because this was a cross-sectional study, a causal association between eating habits, the quality of the diet, and overweight or obesity cannot be proven. Additionally, these findings cannot be generalized to all African students or all African migrant students in China because the sample size was restricted to only African students in Nanjing and African students in specific universities from only three African countries. Finally, the sampling technique was convenient but could not represent the entire population.

## 5 Conclusion

Different nutritional issues affect migrant students in Nanjing compared to their fellow citizens in their home nations. While various fruits and vegetables are absent from the diet of the non-migrants, the Nanjing migrant students tend to engage in more Westernized diets, including deep-fried snacks, sugar-sweetened beverages, processed meat, high-fat dairy, and ice cream, in addition to eating late at night, skipping meals, especially breakfast, and eating once or twice daily. Furthermore, the African migrant students were more likely to be overweight or obese than the non-migrant students due to these unhealthy eating habits, such as eating late at night, eating twice daily, and consuming red meat, processed meats, refined grains, baked goods, sweets, and ice cream. Controlling the what (Western diet and nature of late-night meals) and when of eating can drastically reduce their influence on obesogenic condition formation in African migrant students in China and elsewhere.

## Data availability statement

The original contributions presented in the study are included in the article/supplementary material, further inquiries can be directed to the corresponding author.

## Ethics statement

The requirement of ethical approval was waived by the Nanjing Medical University, School of Public Health. The studies were conducted in accordance with the local legislation and institutional requirements. The participants provided their written informed consent to participate in this study.

## Author contributions

AW: Writing—original draft, Data curation, Methodology, Supervision, Conceptualization, Formal analysis, Project administration, Validation, Investigation, Funding acquisition. MW: Data curation, Investigation, Writing—review & editing. CP: Investigation, Writing—review & editing, Data curation, Software. OA: Investigation, Writing—review & editing. CB: Investigation, Writing—review & editing, Methodology. BA: Investigation, Writing—review & editing, Data curation. SY: Investigation, Methodology, Writing—review & editing. DA: Writing—review & editing, Data curation, Methodology, Project administration, Visualization. SB: Data curation, Investigation, Software, Writing—review & editing. TO: Data curation, Investigation, Writing—review & editing. MZ: Investigation, Validation, Writing—review & editing. YZho: Validation, Visualization, Writing—review & editing. YF: Software, Validation, Writing—review & editing. YZha: Software, Visualization, Writing—review & editing. TW: Validation, Visualization, Writing—review & editing. QF: Conceptualization, Funding acquisition, Project administration, Resources, Supervision, Writing—review & editing.
